# A Compend of the Diseases of the Eye and Refraction

**Published:** 1900-01

**Authors:** 


A COMPEND OF THE DISEASES OF THE EYE AND REFRACTION. Including treatment and surgery. By GEORGE M. GOULD, A. M , M. D., formerly Ophthalmologist to the Philadelphia Hospital, etc , and WALTER L PYLE, A. M., M. D., Assistant Surgeon to Wills' Eye Hospital, Philadelphia, etc Second edition, revised and enlarged. Duodecimo, 295 pages with 109 illustrations, several of which are in colors. Philadelphia : P. Blakiston's Son & Co. 1899.

Blakiston's compends have been very popular for years and have seemed to serve a useful purpose to students. This one on the eye is especially valuable, and in its second edition, it has been enlarged and improved by the addition of several new subjects. In its present form, it is brief, fairly comprehensive and reliable.

